# Serosurveillance of viral pathogens circulating in West Africa

**DOI:** 10.1186/s12985-016-0621-4

**Published:** 2016-10-03

**Authors:** Aileen E. O’Hearn, Matthew A. Voorhees, David P. Fetterer, Nadia Wauquier, Moinya R. Coomber, James Bangura, Joseph N. Fair, Jean-Paul Gonzalez, Randal J. Schoepp

**Affiliations:** 1 Diagnostic Systems Division, US Army Medical Research Institute of Infectious Diseases, 1425 Porter Street, Fort Detrick, MD 21702-5011 USA; 2 Statistics Division, US Army Medical Research Institute of Infectious Diseases, Fort Detrick, MD USA; 3 Metabiota, Inc., San Francisco, CA USA; 4 Kenema Government Hospital, Lassa Diagnostic Laboratory, Ministry of Health and Sanitation, Kenema, Sierra Leone; 5 MRI Global, 1330 Piccard Avenue, Rockville, MD USA

**Keywords:** Serosurvey, West Africa, Sierra Leone, Lassa, Ebola, Marburg, Rift Valley fever, Crimean-Congo, Alphavirus, Flavivirus, Prevalence, Antibodies, IgG, MAGPIX, Luminex

## Abstract

**Background:**

Sub-Saharan Africa is home to a variety of pathogens, but disease surveillance and the healthcare infrastructure necessary for proper management and control are severely limited. Lassa virus, the cause of Lassa fever, a severe hemorrhagic fever in humans is endemic in West Africa. In Sierra Leone at the Kenema Government Hospital Lassa Diagnostic Laboratory, up to 70 % of acute patient samples suspected of Lassa fever test negative for Lassa virus infection. This large amount of acute undiagnosed febrile illness can be attributed in part to an array of hemorrhagic fever and arthropod-borne viruses causing disease that goes undetected and untreated.

**Methods:**

To better define the nature and extent of viral pathogens infecting the Sierra Leonean population, we developed a multiplexed MAGPIX® assay to detect IgG antibodies against Lassa, Ebola, Marburg, Rift Valley fever, and Crimean-Congo hemorrhagic fever viruses as well as pan-assays for flaviviruses and alphaviruses. This assay was used to survey 675 human serum samples submitted to the Lassa Diagnostic Laboratory between 2007 and 2014.

**Results:**

In the study population, 50.2 % were positive for Lassa virus, 5.2 % for Ebola virus, 10.7 % for Marburg virus, 1.8 % for Rift Valley fever virus, 2.0 % for Crimean-Congo hemorrhagic fever virus, 52.9 % for flaviviruses and 55.8 % for alphaviruses.

**Conclusions:**

These data exemplify the importance of disease surveillance and differential diagnosis for viral diseases in Sierra Leone. We demonstrate the endemic nature of some of these viral pathogens in the region and suggest that unrecognized outbreaks of viral infection have occurred.

## Background

Sierra Leone is plagued by a large array of human diseases, but insufficient healthcare infrastructure has resulted in many being unrecognized and uncontrolled. Diseases are often only reported as outbreaks and epidemics, despite their consistent presence in the community. A notable exception is Lassa virus (LASV), which is endemic to Sierra Leone and neighboring countries, and causes Lassa fever (LF), a severe viral hemorrhagic fever that can have a case fatality rate as high as 69 % [1–4]. In eastern Sierra Leone, Kenema Government Hospital (KGH) has a designated LF ward where patients suspected of LASV infection can be isolated and treated. The Lassa Diagnostic Laboratory supports the ward and regional medical facilities and receives approximately 500–700 suspected LF samples annually [1, 5]. Of the submitted samples, only 30–40 % can be attributed to LASV infection, indicating significant disease resulting from other unidentified pathogens. Studies on acute undiagnosed samples from KGH found evidence of hemorrhagic fever and arthropod-borne virus infections including Ebola virus (EBOV), Marburg virus (MARV), Rift Valley fever virus (RVFV), yellow fever virus (YFV), dengue virus (DENV), West Nile virus (WNV), Chikungunya virus (CHIKV), and O'nyong-nyong virus (ONNV) [5, 6], however the disease burden on the human population is unknown. To better define the virus infections over time we undertook a seroprevalence survey of 675 human samples collected at the KGH Lassa Diagnostic Laboratory from suspected LF patients between 2007 and 2014 to detect immunoglobulin G (IgG) antibodies against an array of hemorrhagic fever and arthropod-borne viruses.

Utilizing a multiplex assay on the magnetic bead-based MAGPIX® system (Luminex, Austin, TX), we previously demonstrated increased sensitivity of the MAGPIX® detection platform over enzyme-linked immunosorbent assays (ELISA) for both LASV and EBOV antigen and antibody detection (Satterly et al., submitted). Here, we use the MAGPIX® system to detect and identify virus-specific IgG antibodies against LASV, EBOV, MARV, RVFV, Crimean Congo hemorrhagic fever virus (CCHFV), and pan-assays for flaviviruses and alphaviruses, in an attempt to better define the disease burden on the population over time.

## Methods

### Human samples

The Lassa Diagnostic Laboratory receives blood samples from suspected LF patients and their contacts primarily from Sierra Leone, with occasional samples from Liberia and Guinea. A total of 675 serum samples from suspected LF patients and contacts collected between 2007 and 2014 were tested for IgG antibodies at the Lassa Diagnostic Laboratory and the Diagnostic Systems Division at USAMRIID. Whole blood samples were centrifuged at 200xg, serum collected, and stored at -80 °C until testing. Research on human subjects was conducted in compliance with US Department of Defense, federal and state regulations. All data were gathered and human subject research was conducted under an USAMRIID institutional review board protocol (no. HP-09-32).

### Viral antigen

Viruses used for production of MAGPIX® antigenic materials included LASV Josiah [7, 8]; EBOV Mayinga [9]; MARV Musoke [10]; RVFV ZH 501 strain [11]; CCHFV IbAr10200 [12]; DENV-2 New Guinea C [13, 14]; WNV NY99 [15]; YFV 17D [16]; and CHIKV B8635. All viruses were propagated at the appropriate biological safety level (BSL), either BSL-3 or BSL-4. Briefly, the viruses were grown in appropriate continuous cell lines until cytopathic effects were observed in 50 to 75 % of the cells. Tissue culture supernatants (TCS) were clarified by centrifugation, inactivated by treatment with 0.3 % beta-propiolactone, aliquoted, and stored at -70 °C. Virus infected TCS was inactivated by gamma-irradiation (3 x 10^6^ rads) and safety tested to ensure inactivation. Optimal dilutions of antigens were determined by checkerboard titrations against virus specific antibodies. Mock TCS antigens used as negative controls were prepared as described above using uninfected cell monolayers.

### MAGPIX® assay development

To develop a multiplexed IgG detection assay, we first chose monoclonal antibodies (MAbs) based on target specificity and exclusivity. In the case of the pan-assays, we considered inclusivity of multiple virus family members. Initial antibody selection was based on ELISA checkerboard assessments with the viruses of interest. MAbs with strong affinity for their target were coupled to MAGPIX® magnetic microspheres at a concentration of 4 μg antibody/1 X 10^6^ beads using the Luminex Antibody Coupling Kit (catalog #4050016) according to manufacturer’s instructions. They were then optimized for MAGPIX® IgG detection, and tested for exclusivity against the additional viral targets. Pan-assays utilized MAbs with the greatest cross-reactivity to the virus family. MAbs coupled to MAGPIX® microspheres and used in MAGPIX® IgG detection assays were as follows: anti-LASV glycoprotein complex (GP) L52-85-6-BG12; anti-LASV nucleoprotein (NP) L52-2159-15; anti-EBOV glycoprotein (GP) Z-DA06-AH05; anti-EBOV viral protein 40 (VP40) M-HD6-A10A; anti-MARV VP40 3MI2; anti-RVFV nucleocapsid (NC) R1-P6F6-6-2-2; anti-CCHFV nucleocapsid (N) CCII 12G10-2-2A; anti-YFV envelope (E) YF-211-4E11 (pan-flavivirus capture capable of detecting IgG antibodies to DENV, WNV, YFV, Japanese encephalitis virus (JEV), and tick-borne encephalitis virus (TBEV)) ([17], unpublished data); and anti- Sindbis virus (SINV)/Semliki forest virus glycoprotein E1 (E1) SLK42 (pan-alphavirus capture capable of detecting IgG antibodies SINV, CHIKV, ONNV, Venezuelan equine encephalitis virus (VEEV), Western equine encephalitis virus (WEEV), and Eastern equine encephalitis virus (EEEV) ([18], unpublished data). Virus-specific sets of microspheres were combined into a multiplex assay that could detect several targets in a single sample well for IgG detection.

### MAGPIX® IgG detection

Nine distinct assays detecting IgG antibodies against LASV-GP, LASV-NP, EBOV-GP, EBOV-VP40, MARV-VP40, RVFV-NC, CCHFV-N, YFV-E (pan-flavivirus), and SINV-E1 (pan-alphavirus) were combined in a multiplexed IgG detection assay used to test the 675 human sera samples. Microspheres, 2000 of each of the nine sets, were combined into all wells of 96-well plates; to which either mock TCS or a mixture of viral infected TCS was added. The plates were covered, incubated at room temperature (RT) for 1 h with shaking at 400–500 rpm, and washed three times using a magnetic plate separator. Patient serum was diluted 1:100 and added to wells in triplicate. The plates were covered and incubated at room temperature for 1 h with shaking. The plates were washed, and anti-human IgG-R-Phycoerythrin (Sigma, St. Louis, MO) was added at a 1:100 dilution. The plates were covered, incubated at room temperature for 30 min, washed, and read on a MAGPIX® instrument. The median fluorescence intensity (MFI) of each bead set in each well was obtained. Throughout the assay, the total volume for each step was 50 μl. All dilutions and washes were with 100 μl phosphate-buffered saline with 0.02 % Tween-20.

### Statistical analysis of MAGPIX® results

Assay results were log transformed prior to analysis. For each sample, the z-score was calculated by the mean difference between the log transformed sample replicates in viral infected TCS and the log transformed sample replicates in uninfected TCS, divided by the standard error of the difference. The MFI variance on the log scale was found to be homogenous within each test, and an appropriate pooled variance estimate was taken across each test in calculating standard errors. Results from multiple tests were collected from a single well, and the data for each test were analyzed separately. This analysis step was carried out with a generalized linear model having identity link and normal distribution, as provided in the SAS® GENMOD procedure [19]. Results that had a conservative z-score of at least three standard errors above zero were considered a positive test. Samples testing positive for one or more targets of a single virus was considered positive for the virus; for example, if a sample tested positive for LASV-NP and negative for LASV-GP, it was considered positive for LASV. Prevalence was calculated using the dichotomization obtained at the three standard errors cut point. Although 675 serum samples were assayed, test results with readings from less than ten beads per well were considered unreliable and therefore excluded from analysis; therefore, the total number of samples analyzed for each test varied.

## Results

### Prevalence of anti-viral IgG antibodies

A total of 675 serum samples submitted to the Lassa Diagnostic Laboratory in Kenema, Sierra Leone were subjected to serological testing. Samples were tested for IgG antibodies to specific viral targets and results are presented in Table 1. Of the data collected, 50.2 % (*n* = 328) of samples had detectable antibodies to LASV, consistent with previous estimates of 8–52 % in the area [20]. Antibodies against EBOV were detected in 5.2 % (*n* = 35) of the sample population, MARV in 10.7 % (*n* = 71), RVFV in 1.8 % (*n* = 12), and CCHFV in 2.0 % (*n* = 13). Detectable antibodies to one or more flaviviruses were demonstrated in 52.9 % (*n* = 330) of the samples, and 55.8 % (*n* = 373) had detectable antibodies to one or more alphaviruses. Of the seven distinct viruses (including the two pan-assays), the mean number of positive tests was 1.8, with 26.2 % of individuals testing positive for 3 or more distinct viruses, as shown in Table 2. A more accurate number is likely higher, considering the pan-alphavirus and pan-flavivirus tests detect multiple exposures.

**Table 1:** Seroprevalence of each target virus among samples obtained from suspected LF patients. Number of samples testing positive, total number tested, and percent tested positive by MAGPIX

Observed Prevalence Rates	
Analyte	Positive/total tested (%)
LASV	328/654 (50.2 %)
EBOV	35/672 (5.2 %)
MARV	71/663 (10.7 %)
RVFV	12/667 (1.8 %)
CCHFV	13/641 (2.0 %)
Pan-flavivirus	330/624 (52.9 %)
Pan-alphavirus	373/668 (55.8 %)

**Table 2:** Distribution of number of positive tests identified per sample, out of seven distinct tests (LASV, EBOV, MARV, RVFV, CCHFV, Pan-alphavirus, and Pan-flavivirus). Samples that did not have valid results for all 7 distinct tests are listed as not applicable (N/A)

Frequency of Positive Tests per Sample		
Number of positive tests per sample	Frequency (n)	Percent (%) of total samples
0	109	16.15
1	138	20.44
2	174	25.78
3	137	20.30
4	29	4.30
5	6	0.89
6	4	0.59
7	1	0.15
N/A	77	11.41

### Longitudinal assessment of prevalence

A longitudinal assessment to investigate chronological trends in positive IgG antibody rates was performed and presented in Table 3. Surprisingly, MARV antibodies were detected in 23 % of samples tested from 2008, suggesting a possible unrecognized outbreak of the virus in the area. Of additional note is the large increase in RVFV in 2014, and the fluctuations in alphavirus positive rates. It should be noted that year 2009 had only one representative sample and was therefore not included in the table below.

**Table 3:** Observed seropositive rates are reported in percent of total samples from that year for each pathogen. There was only one representative sample from the year 2009, therefore statistics for that year are not included in the chart

		Seroprevalence rate (% of total each year)						
Year	Total samples	LASV	EBOV	MARV	RVFV	CCHFV	Pan-flavivirus	Pan-alphavirus
2007	51	41	0	8	0	0	49	61
2008	151	57	3	23	1	1	38	68
2010	195	51	7	7	1	2	67	65
2011	153	36	5	6	3	3	39	32
2012	66	67	10	13	1	1	64	63
2013	41	61	2	0	0	2	73	49
2014	19	37	0	0	11	5	47	26

## Discussion

Knowledge of the diseases circulating in a region is paramount for proper diagnosis, care and treatment of patients, and ultimately a reduction of overall disease burden. Sierra Leone and the surrounding areas suffer from numerous viral diseases, but surveillance and diagnostic capabilities fall short of the need for effective disease control. Moreover, as demonstrated by the recent EBOV outbreak in West Africa, differential diagnosis among febrile illnesses and knowledge of their prevalence is of great importance for timely and efficient management of patients and outbreak prevention. To more accurately define the disease burden on the population, we applied a multiplexed serological assay to screen a panel of 675 serum samples from Sierra Leone to identify some of the arthropod-borne and hemorrhagic fever viral burden in the region.

The MAGPIX platform, a multiplexed, bead-based system, can efficiently collect valuable disease burden information where it is not routinely monitored. Customization and optimization of multiple assays in a single well permitted testing for each target of interest in less time and with less effort than other methods such as ELISA. In our study population, 26.2 % were positive for exposure to at least three viruses. Since we investigated only IgG antibodies, the results represented the infection of individuals over their lifetimes and not a single point in time, therefore it was expected to find antibodies to multiple targets in a single individual. This further highlights the severe extent of public health risk of virus infection for the population.

Among suspected LF patients we found LASV seroprevalence to be 50.2 %, which is consistent with estimates ranging from 8–52 % throughout the country and the hyper-endemic region in eastern Sierra Leone, where KGH is located [20]. EBOV and MARV seroprevalence were 5.2 % and 10.7 %, respectively. A recent study estimated seroprevalence of filoviruses to be 22 % in the area, and an IgM survey from the same hospital reported a 9 % acute EBOV infection rate between 2006 and 2008 [5, 6]. Considering the estimated fatality rates of EBOV and that we are observing latent immunity only present in survivors, the 5.2 % prevalence seen here correlates appropriately with the 9 % observed acute cases during the time period of sample collection. In our study, EBOV infections were demonstrated in the population as early as 2008 and earlier by others [5], indicating circulating virus throughout the time period that may be the result of a reservoir maintaining EBOV in the environment. We found the overall seroprevalence of MARV to be 10.7 % and present as early as 2007; although there are no records of MARV outbreaks or disease in this region, it was found retrospectively in 3.6 % of acute samples from KGH dating from 2006–2008, suggesting an increase in exposures occurred in the last 7 years. There is a notable jump in MARV to 23 % seroprevalence in 2008, suggesting a possible unrecognized outbreak of the MARV in the area. Since no increase in VHF was noted, it might be that the MARV circulating is a less virulent strain.

RVFV is a bunyavirus known to circulate throughout sub-Saharan Africa, mainly among livestock and *Aedes* species mosquitos, with sporadic outbreaks of human disease. While the climate and epizootic factors are well studied in East and South Africa, it has been postulated that there are other factors supporting endemic sustainability in West Africa [21–23]. Here we report a seroprevalence of 1.8 %, and observed a notable increase in prevalence from 1–3 % in 2007–2013 to 11 % in 2014, suggesting a substantial and recent increase in its circulation. Another bunyavirus, CCHFV, is known to occur throughout Africa, Asia and Europe in animals and ticks. Human infection, albeit rare, is severe and usually associated with livestock contact [24–27]. We detected low numbers of CCHFV exposure (*n* = 13, 2.0 %) in our sample population, even though it was not detected in other recent studies [5, 6]. The relatively low but consistent prevalence of antibodies to RVFV and CCHFV may suggest the presence of reservoirs or continual reintroduction to the region resulting in low, but consistent levels of human infection.

Flaviviruses and alphaviruses cause a large number of infections and disease throughout West Africa. Serological data is difficult to interpret since there is significant cross-reactivity among viruses within their respective families. Generally, antibodies to a specific virus must be distinguished by plaque reduction neutralization tests. It is known that multiple alphaviruses and flaviviruses circulate in this region, some described and others possibly undiscovered, but our interests were in the prevalence of the overall disease attributed to each group. Therefore given the number of samples tested here and the desire for broad and complete surveillance, we developed two pan-assays intended for the widest possible coverage of flavivirus and alphavirus species. Antibody prevalence rates were high for viruses of both families; combined prevalence of flavivirus and alphavirus antibodies were 52.9 % and 55.8 %, respectively. There are limited data on the seroprevalence to flaviviruses in Sierra Leone. A survey by Boisen et al. (*n* = 77) from Kenema Government Hospital revealed 45 % seroprevalence to DENV and 54 % to WNV [6]. Studies in the neighboring countries of Guinea, Nigeria, and Cameroon report a range of seroprevalence to flaviviruses including YFV (27–43 %), DENV-2 (12–45 %) and WNV (7–49 %) [28–30], each of which is detectable in our pan-assay and likely represent significant portions of the overall seroprevalence. It is also possible that YFV vaccination may have increased the prevalence of flaviviruses recorded here; distribution of the vaccine in Sierra Leone before and during the time period of the samples examined here is estimated to have a population coverage of 75 % or more [31, 32]. Similar to the flaviviruses, there is limited information on the prevalence of alphavirus antibodies in Sierra Leone. Boisen et al. reported in the same survey of 77 KGH serum samples a prevalence to CHIKV of 27 % [6]. The 55.8 % prevalence of alphavirus antibodies that we report here is similar to estimates of CHIKV and ONNV in nearby Cameroon where approximately 47 % of healthy adults tested positive for CHIKV and/or ONNV (with noted overlap) [30]. Our longitudinal data analysis revealed a high prevalence of alphaviruses prior to 2010 (>60 %), which may be in part due to ONNV and CHIKV outbreaks known to occur in Guinea in 2003 [33] and 2006 [34]. Additionally, a spike in 2012 and a subsequent decrease in the following years correlates with a reported CHIKV outbreak in Sierra Leone in 2012, identified in a hospital only 60 kilometers from KGH [35]. The high rates of both flaviviruses and alphaviruses seen here, combined with identified recurring outbreaks of CHIKV and ONNV, highlight the range of endemic viruses and their significant impact on the limited medical infrastructure.

Retrospective studies have limitations by their very nature. In this study the samples tested had a bias for subjects that 1) were willing to seek help from the hospital, and 2) had at some point presented with symptoms resembling LF. An individual’s presentation at the hospital may indicate they are more likely to be exposed to pathogens via factors in their lifestyle, geographic location, or workplace environment.

## Conclusions

The results of this study indicate that in addition to LASV, there is a significant presence of filoviruses, bunyaviruses, flaviviruses and alphaviruses actively circulating in the Sierra Leone region over time. Some virus pathogens are consistently circulating over the entire 7-year timespan tested, which suggests many are endemic, being maintained in the region in natural reservoirs. We found indications of possible unrecognized outbreaks of infection or subclinical exposure that add to the disease burden in the human population. Sierra Leone and other regions where the public health burden of these pathogens is insufficiently evaluated need improved pathogen surveillance. Multiplexed and bead-based platforms such as MAGPIX can provide an efficient, customizable surveillance method to evaluate pathogen presence, spread and risk.

## Abbreviations

BSL: Biological safety level
CHIKV: Chikungunya virus
DENV: Dengue virus
YFV-E: Yellow fever virus envelope protein
SINV-E1: Sindbis/Semliki forest glycoprotein E1
EBOV: Ebola virus
EEEV: Eastern equine encephalitis virus
ELISA: Enzyme-linked immunosorbent assay
EBOV-GP: Ebola virus glycoprotein
LASV-GP: Lassa virus glycoprotein
IgG: Immunoglobulin G
JEV: Japanese encephalitis virus
KGH: Kenema Government Hospital
LASV: Lassa virus
LF: Lassa fever
MAbs: Monoclonal antibodies
MARV: Marburg virus
MFI: Median fluorescence intensity
CCHFV-N: Crimean-Congo hemorrhagic fever virus nucleocapsid
N/A: Not applicable
RVFV-NC: Rift Valley fever virus nucleocapsid
LASV-NP: Lassa virus nucleoprotein
ONNV: O'nyong-nyong virus
RT: Room temperature
RVFV: Rift Valley fever virus
SINV: Sindbis virus
TBEV: Tick-borne encephalitis virus
TCS: Tissue culture supernatants
VEEV: Venezuelan equine encephalitis virus
VP40: Viral protein 40
WEEV: Western equine encephalitis virus
WNV: West Nile virus
YFV: Yellow fever virus
